# Treatment adherence and facilitator characteristics in a community based pediatric weight control intervention

**DOI:** 10.1186/1479-5868-11-17

**Published:** 2014-02-13

**Authors:** Elissa Jelalian, Gary D Foster, Amy F Sato, Kristoffer S Berlin, Cynthia McDermott, Deborah Sundal

**Affiliations:** 1 Bradley Hasbro Children’s Research Center, Alpert Medical School of Brown University, Providence, RI 02903, USA; 2 Center for Obesity Research and Education, Temple University, Philadelphia, PA, USA; 3 Department of Psychology, Kent State University, Kent, OH, USA; 4 The University of Memphis, Memphis, TN, USA; 5 YMCA of Greater Providence, Providence, RI, USA; 6 UnitedHealth Group for Health Reform and Modernization, UnitedHealth Group, Minneapolis, MN, USA

**Keywords:** Obesity, Pediatric, Intervention, Community, Facilitator

## Abstract

**Background:**

There is a pressing need to develop effective and broadly accessible interventions to address pediatric obesity. An important dimension in translating interventions to community settings is evaluating the fidelity with which the intended treatment is delivered and the level of facilitator needed to deliver the intervention with efficacy.

**Purpose:**

The primary objectives of this study were to: 1) provide descriptive information regarding adherence to protocol and non-specific facilitator characteristics (e.g. interpersonal characteristics, group management skills) within the context of a community based pediatric weight control intervention delivered by paraprofessionals; and 2) examine the relationships among facilitator adherence and characteristics and rate of change in percent overweight demonstrated by youth over the course of the 24-week intervention.

**Methods:**

The intervention was conducted between February and September of 2011. Children (6–16 years) and parents completed primary outcome measures at baseline, 12, and 24 weeks (i.e. end of treatment). A 2-part rating form was developed to assess facilitator adherence to weekly content and general provider characteristics at two different time points during the intervention.

**Results:**

Youth participating in this study were on average 11.3 years old (SD = 2.8), with most being under the age of 13 years (74.2%). Over half were female (54.8%) and over two-thirds were White (68.4%). On average, facilitators adhered to 96.0% (SD = 5.2%) of the session content at Time 1 and 92.6% (SD = 6.8%) at Time 2. Higher Content Adherence at Time 1 and Time 2 were associated with greater loss in percent overweight.

**Conclusions:**

Our data suggest that paraprofessionals without prior expertise in pediatric weight control can be trained to successfully deliver an intervention that is evidence based and incorporates behavioral and educational components. These findings need to be considered in light of some limitations, including the fact that facilitator domains were assessed with a modification of a standardized tool and we did not obtain inter-rater reliability of observations. These limitations not withstanding, investing time in training facilitators to adhere to a given protocol is critical and may be of higher priority than focusing on more general facilitator characteristics.

## Background

Pediatric obesity is an epidemic in the United States, with approximately one third of youth identified as overweight or obese [1,2]. Obesity in youth is associated with a number of health risks, including elevations in cholesterol, diastolic and systolic blood pressure, triglycerides, and fasting insulin levels as well as increased risk for type 2 diabetes [3,4]. Furthermore, obesity in children tracks into adulthood, which in turn is related to increased rates of morbidity and mortality [5]. In addition to health risks, youth who struggle with excess weight are at greater risk for a range of psychosocial difficulties including decreased self-esteem, challenges in peer relationships [6,7], and impairments in health related quality of life (HRQOL) [7,8].

Family-based behavioral weight control programs have demonstrated efficacy in the treatment of overweight children between the ages of 8 and 12 years, with some promise regarding long-term outcomes. While effective, these interventions have been administered through carefully controlled clinical trials [9,10] or delivered in specialized/tertiary clinical settings affiliated with academic medical centers. As a result, they are available for only a small percentage of children and adolescents struggling with excess weight. The need for identifying alternative, more broadly accessible delivery systems for these interventions increased with the USPSTF recommendation that primary care providers refer youth to behavioral intervention programs [11].

There are very few examples of interventions to assist children and adolescents who are already overweight/obese that are delivered in community settings and available to a greater segment of the population. We are aware of only three studies explicitly designed to examine effectiveness of pediatric obesity interventions in a community setting. Project STORY [12] examined the utility of Cooperative Extension Service offices in rural settings for delivery of a weight control intervention for children 8–12 years. Children assigned to both a family based and parent only condition demonstrated greater decrease in BMI z-score than those assigned to wait list control at 10-month assessment. A second effectiveness study involved examination of the MEND Program developed in the United Kingdom [13]. Children between 8–12 years randomized to receive the intervention demonstrated significant decreases in BMI (-1.2 kg/m^2^) and waist circumference (-4.1 cm) compared to those assigned to a delayed treatment control. More recently, the JOIN program, developed by UnitedHealth Group and delivered through the YMCA, was shown to significantly decrease percent overweight among school age children [14].

While these interventions have been effective, little is known regarding the fidelity of treatment implementation, including adherence to treatment protocols and facilitator characteristics. Such information is critical for scalability of health change interventions, as it has direct implications for interpretation and potential generalizability of study findings. In a recent review, only 5% of more than 75 weight control studies reported on the extent to which the intervention content was delivered as intended [15]. Attention to treatment fidelity is also highlighted by the NIH Behavior Change Workgroup, which includes recommendations for defining, evaluating, and enhancing treatment fidelity in behavioral interventions [16]. In addition to examining the extent to which the intervention was delivered as intended, the Workgroup further emphasized the importance of assessing provider characteristics (e.g. warmth) [16].

The importance of treatment fidelity is further highlighted in the RE-AIM framework [17], a model helping to increase the external validity and public health impact of health interventions. Within this framework, “Implementation” is a key domain that includes the extent to which project staff delivered an intervention as intended [18]. Historically, both weight control prevention and intervention studies have provided minimal information regarding the consistency with which interventions are delivered and potential differences of treatment efficacy across interventionists [15,19]. We are not aware of any previous research that has examined the combination of treatment fidelity and provider characteristics as related to outcomes for pediatric behavioral weight control interventions. Assessment of fidelity is particularly important in the context of development and implementation of a scalable intervention, given the absence of a tightly controlled environment and the broad scale implications for dissemination.

We had the opportunity to examine treatment fidelity and facilitator characteristics in the context of a recently delivered 24-week community based pediatric weight control program for youth ages 6–17 years old. The JOIN program [14] involved a collaboration between UnitedHealth Center for Health Reform and Modernization and the YMCA of Greater Providence to deliver an empirically supported family based behavioral weight control intervention. Intervention components from evidence based treatments were modified to reduce cost and increase scalability. Specific modifications included: intervention delivery by YMCA facilitators with no previous training in pediatric obesity, combined child and parent groups, and 12 in-person sessions (efficacy trials typically include a greater number of sessions), with 12 additional sessions conducted by parents at home. Findings from the initial pilot were recently published and indicated a 4.3% reduction in percent overweight for children between 6–12 years, with a smaller decrease for adolescents [14]. These results have significant implications for continued development of scalable interventions to address pediatric obesity that can be delivered in community settings. To be effective, such interventions will require replicable models for training paraprofessional facilitators as well as identification of key facilitator characteristics that are related to positive outcomes.

The purpose of this study was to examine two key components of treatment fidelity: facilitator adherence to treatment content and non-specific facilitator characteristics in the context of a community based pediatric weight control intervention. Primary study objectives were to: 1) provide descriptive information regarding adherence to the treatment protocol and non-specific facilitator characteristics (e.g. interpersonal characteristics, group management skills); and 2) examine the relationships among facilitator adherence and characteristics and rate of change in percent overweight demonstrated by youth over the course of the 24-week intervention.

## Methods

Children and parents completed primary outcome measures at three evaluation points, baseline, 12 weeks, and 24 weeks (i.e. end of treatment).

### Anthopometrics

Body weight was measured on calibrated scales (Detecto Model 6129, Cardinal Scale Manufacturing Company, Webb City, MO) with participants wearing light clothing and no shoes. Height was measured using a stadiometer (Seca 217 Mobile Stadiometer, Seca, Hamburg, Germany). Body mass index was calculated as weight (kg) divided by height (m)^2^. Percentage overweight was calculated as the percentage over the median BMI for age and gender.

### Facilitator ratings

A total of 16 groups (12 child, 4 adolescent) were conducted at eight different YMCA sites in the Providence, RI area. Nine of the groups were based in urban locations, with seven in suburban/rural settings. Sessions were led by nine YMCA-based facilitators, 7 of whom had Bachelor’s degrees and 2 of whom had Masters degrees. All of the facilitators had some experience supervising groups and 7 had experience with behavioral coaching. None of the facilitators had experience with pediatric obesity, although 3 of them had prior experience with adult weight control. Three of the nine facilitators led both child and adolescent groups.

YMCA facilitators received a total of 4.5 days of in-person training with experienced behavioral weight control interventionists (Drs. Foster and Jelalian), including 2 ½ days prior to start of the program and 2 1-day sessions during the course of the intervention. The initial training session included an orientation to the program, review of behavioral weight control strategies with children and adolescents, recommendations for managing group interactions, review of the first several lesson plans, and information regarding involvement in research and administrative oversight. Approximately 1 ½ days were focused on clinical content. The additional 2 full-day trainings focused exclusively on review of treatment materials and role-plays of lesson content. Throughout treatment delivery, facilitators also participated in 1-hour weekly telephone based supervision meetings to facilitate adherence. Approximately 75% of each hour long call was devoted to treatment related issues, including review of upcoming content, a question and answer period, and concerns regarding specific participants. The remaining time was dedicated to logistical issues such as use of the program tracking tool to document weekly attendance and weight. This study was approved by the New England Institutional Review Board.

The intervention included a total of 12 face-to-face sessions conducted at the YMCA and 12 briefer (i.e. 10–15 minute) home-based sessions led by parents. Home sessions were designed to last 10–15 minutes and followed the general format of a parent and child reviewing progress with behavioral goals and the parent briefly introducing new content provided to them by the YMCA facilitator. Face to-face sessions were held across the 24-week intervention at weeks: 1, 2, 3, 4, 8, 10, 12, 14, 16, 18, 20, and 24. Each facilitator was observed twice during the 24-week intervention. The “time 1” observation was conducted during session 1, 2, 3, or 4 of the face-to-face sessions. The “time 2” observation was conducted during session 8, 10, 12, 14, 16, 18, or 20 of the face-to-face sessions. All facilitators except for one were observed once at time 1 and once at time 2. This facilitator was observed only once because the group was discontinued. For the three facilitators who conducted both child and teen groups, observations were made for one of the child and adolescent groups, resulting in a total of 12 observations at each of two time points. All observations were conducted by one of two observers, a postdoctoral fellow in pediatric psychology or a clinical psychologist with expertise in weight control.

A 2-part rating form was developed to assess facilitator adherence to weekly content and general provider characteristics. Weekly content adherence was measured as the extent to which the facilitator covered each of the prescribed topics, with ratings of Yes, No, and Partial. Nine-items evaluated more general provider characteristics such as interpersonal skills and ability to effectively manage group interactions. These items were adapted from a well validated psychotherapy assessment tool [20] and rated on a 5-point likert scale. A final item included assessment of overall expertise (1–3 = “Novice,” 4–6 = “Intermediate,” and 7–9 = “Advanced”).

### Analytic Plan

In order to examine the relationships among facilitator adherence, facilitator characteristics, and rate of change in youth’s percent overweight over the course of the 24-week intervention, random effects growth modeling was conducted using Mplus 6.11 [21]. This approach coupled with robust maximum likelihood estimation allowed for the modeling of change with nested and non-normally distributed data. Robust estimation of standard errors takes into account both non-normality of outcomes and non-independence of observations due to the nesting of study participants across group facilitators. The random effects capture individual differences, in terms of random slopes (i.e., individual differences in the rate of change in percent overweight across the 24 week program) and intercepts (initial percent overweight values). As a necessary first step, an unconditional linear growth model was evaluated to determine whether there was significant variability in youth’s rate of change in percent overweight over the 24 weeks of treatment. Pending significant variability in the rate of change, a second model (Table 1) was evaluated in which youth’s rate of change in percent overweight was predicted by facilitator adherence, facilitator characteristics, percentage of session content covered, and youth’s age and initial percent overweight. This model allowed all predictors to correlate with one another.

**Table 1:** Parameter estimates and inferential statistics for the growth models (N = 155)

**Model 1: unconditional lLinear**	**Estimate**	**S.E.**	**Est./S.E.**	**P-value**
Initial Percent Overweight (POW) with slope (rate of change)	3.502	2.565	1.366	0.172
**Means**				
Initial POW	72.096	3.723	19.365	0.000
Slope	-0.138	0.039	-3.561	0.000
**Variances**				
Initial POW	1123.825	198.105	5.673	0.000
Slope (rate of change)	0.167	0.032	5.156	0.000
Residual variances*				
POW initial	17.158	5.242	3.273	0.001
POW week 12	17.158	5.242	3.273	0.001
POW week 24	17.158	5.242	3.273	0.001
**Model 2: predictors of rate of change**	**Estimate**	**S.E.**	**Est./S.E.**	**P-value**
Initial POW	0.003	0.002	1.617	0.106
Age in months	0.001	0.001	0.672	0.502
Facilitator characteristics (time 1)	0.000	0.010	0.025	0.980
Facilitator characteristics (time 2)	0.064	0.019	3.433	0.001
Expert rating (time 1)	-0.064	0.046	-1.391	0.164
Expert rating (time 2)	-0.082	0.051	-1.615	0.106
Percent content adherence (time 1)	-0.016	0.005	-2.870	0.004
Percent content adherence (time 2)	-0.027	0.009	-3.132	0.002

*residual variances constrained to be equal.

Note. POW = Percent Overweight; Facilitator Characteristics = Sum of the 9 item facilitator characteristics scale.

## Results

### Participants

Participants included 155 children and one parent/guardian per child, and were referred to the program through area pediatricians, school nurses, and postings at local YMCA facilities. Participating youth were on average 11.3 years old (SD = 2.8), with more children under the age of 13 years (74.2%) than adolescents ages 13 and older (25.8%). Over half of children participating in this study were female (54.8%) and over two-thirds were White (68.4%). At baseline, participants had an average BMI of 30.5 (SD = 7.3), a BMI z-score of 2.23 (SD = .41), and percentage overweight of 72.5 (SD = 34.0). The vast majority (91.6%) of youth were obese (≥ 95th BMI%ile) and nearly half (46.5%) met the 99th BMI%ile for age and gender.

### Weight change outcomes

Eighty four percent of the sample (*n* = 130) completed the 24 week assessment, this included 86% of children (99/130) and 78% of adolescents (31/40). Completers were defined as participants who attended at least one group session and were present for the 24-week assessment. Completers and non-completers did not differ on baseline characteristics. We also examined attendance by age group and found that children attended a mean of 8.62 face-to-face sessions and 8.16 home-based sessions, while adolescents attended an average of 9.39 face-to-face sessions and 9.80 home-based sessions. Attendance rates did not differ significantly for children <13 and those ≥ 13 years.

For the overall sample, there was a significant reduction in percent overweight (M = 3.5%) from baseline to 24 weeks. Children (<13 years old) experienced a 4.5% (<.001) reduction in percent overweight from baseline to 24 weeks, while adolescents (≥ 13 years old) did not show a significant reduction (0.2% reduction in percent overweight, p > .05. This may reflect the fact that a number of adolescents had BMIs in excess of 45 and may have been less well suited for a lifestyle intervention. No significant gender effects emerged for change in percent overweight at 24 weeks.

### Descriptive findings

#### Intervention content adherence

An observation of the extent to which group facilitators adhered to intervention content was conducted twice for each of the nine facilitators. On average, facilitators were observed to adhere to 96.0% (SD = 5.2%) of the session content at time 1. Adherence to session content at time 2 was slightly lower, at 92.6% (SD = 6.8%). There were no significant differences on any domains for facilitators who led one group (*n* = 5) compared to those who led more than one group (*n* = 4).

#### Facilitator characteristics and expert facilitator rating

Alpha reliability for the 9-item facilitator rating scale was 0.86 both at time 1 and time 2. Average ratings on the facilitator rating scale were 4.04 (SD = 0.61) at time 1 and 4.14 (SD = 0.47) at time 2. Facilitator rating sum scores at times 1 and 2 and average scores on each of the nine individual facilitator rating scale items are presented in Table 2. Overall facilitator competence in delivering the JOIN intervention content, as measured by the expert facilitator rating, was in the Intermediate expertise category both at time 1 (M = 5.92, SD = 1.56) and at time 2 (M = 6.09, SD = 1.30) at time 2.

**Table 2:** Facilitator characteristics at Time 1 and Time 2

	**Time 1 ratings**	**Time 2 ratings**
	**(M, SD)**	**(M, SD)**
	**n = 12**	**n = 11**
Item 1: Transition smoothly between activities	3.83, 0.83	3.91, 0.70
Item 2: Use open-ended questions as a means of encouraging participants to talk	3.75, 1.06	4.18, 0.75
Item 3: Encourage children and parents involvement with discussion and activities	4.03, 0.90	4.27, 0.79
Item 4: Encourage group members to develop their own solutions to problems and prompt use of problem-solving when appropriate	4.08, 0.67	4.18, 0.60
Item 5: Manage participant behavior effectively to minimize disruptions to group process (e.g. maintain focus, help get participants back on track)	4.42, 0.51	4.00, 0.45
Item 6: Demonstrate strong interpersonal skills by expressing warmth, genuineness, and enthusiasm while delivering content	4.42, 0.67	4.64, 0.67
Item 7: Reinforce participant efforts by using praise and minimal encouragements (e.g. “uh-huh”, “okay”)	4.17, 0.83	4.00, 0.77
Item 8: Support participants by acknowledging progress toward weekly behavioral goals	3.70, 1.06	4.00, 0.77
Item 9: Deliver intervention without relying too much on written manuals (e.g. not reading)	3.92, 0.90	4.09, 0.54
Total sum of facilitator characteristics (sum across nine items)	35.75, 5.80	37.27, 4.22
Facilitator characteristics (average across nine items)	4.04, 0.61	4.14, 0.47
Expert facilitator rating	5.92, 1.56	6.09, 1.30

Note: Items 1–9 rated on a 5-point scale where 1 = not at all and 5 = advanced. Expert facilitator rating on a 9-point scale were 1–3 = novice, 4–6 = Intermediate, and 7–9 = Advanced.

Results from the first growth model are presented in Table 1 (model 1). Youth (on average) began at 72.10% overweight (intercept) and their percent overweight decreased significantly by 0.14 per week over the 24 week program. Youth’s rate of change was unrelated to their initial percent overweight. Table 1 also reveals significant individual differences in youth’s initial percent overweight and rate of change, which permitted further investigation of predictors of change.

A second model (Table 1) was evaluated in which youth’s rate of change in percent overweight was predicted by facilitator characteristics, facilitator expert rating, percentage of session content covered, and youth’s age and initial percent overweight. Of the predictors presented in Table 1, three were significant: Facilitator Characteristics (time 2), Content Adherence (time 1), and Content Adherence (time 2). More specifically (holding all other predictors constant), a one unit increase in Content Adherence at time 1 was associated with an additional 0.016 weekly reduction in percent overweight. Similarly, a one unit increase in Content Adherence at time 2 was associated with an additional 0.027 weekly reduction in percent overweight over the course of the trial. Unexpectedly, higher ratings of Facilitator Characteristics at time 2 were associated with (0.06) less loss in percent overweight during the course of the 24 week program. Facilitator Characteristics (time 1), expert rating (time 1), expert rating (time 2), youth’s age, and initial percent overweight were not related to youth’s rate of change in percent overweight. Table 3 presents the means, standard deviations, and correlations among study variables (taking into account non-normality and nesting).

**Table 3:** Correlations among study variables, (off diagonals), and means and (Standard Deviations) corrected for non-normality and nesting

	**1**	**2**	**3**	**4**	**5**	**6**	**7**	**8**	**9**	**10**
1. Age (months)	135.88 (33.60)									
2. Facilitator characteristics (time 1)	0.02	35.99 (4.96)								
3. Facilitator characteristics (time 2)	0.04	0.54*	37.47 (4.36)							
4. Expert rating (time 1)	-0.05	0.88*	0.57*	6.01 (1.39)						
5. Expert rating (time 2)	0.10	0.65*	0.80*	0.74*	6.30 (1.23)					
6. Percent content adherence (time 1)	-0.29	0.44	0.17	0.44	0.39	97.06 (4.57)				
7. Percent content adherence (time 2)	0.25	0.07	0.68	-0.02	0.25	-0.30	91.34 (6.17)			
8. POW (week 1)	0.18	0.03	0.02	-0.04	0.00	-0.03	0.10	72.49 (33.87)		
9. POW (week 12)	0.21	0.02	0.03	-0.04	0.00	-0.05	0.10	0.98*	69.53 (35.01)	
10. POW (week 24)	0.18	0.00	0.04	-0.06	-0.01	-0.07	0.11	0.95*	0.98*	69.34 (37.60)

(*p ≤ 0.05).

Note. POW = Percent Overweight; Facilitator Characteristics = Sum of the 9 item facilitator characteristics scale.

## Discussion

Facilitator training for this community based pediatric weight control intervention, which consisted of approximately 2 ½ days of in person didactics followed by two 1-day booster sessions, and weekly phone supervision, resulted in a high level of adherence in delivering weekly content as intended. It is noteworthy that during both the first and second observations, facilitators exceeded 90% in their coverage of prescribed material. These findings clearly document that paraprofessionals without prior experience in pediatric weight control can be effectively trained to deliver a complex evidenced based weight control intervention.

Overall, YMCA staff also scored highly on ratings of facilitator characteristics (e.g., expressing warmth, genuineness, and enthusiasm) traditionally associated with positive outcomes in the psychotherapy literature [22]. Relatively lower scores were observed on use of open ended questions and attention to behavioral goals, while higher ratings were seen on less specific domains of interpersonal skills and managing group behavior.

To our knowledge, this is the first community based obesity treatment study to include examination of intervention implementation. Previous research suggests variability in treatment fidelity for programs targeting youth health practices (e.g. substance abuse) in a real-world setting [23]. Although not specific to obesity, assessment of fidelity within the context of school-based nutrition education curriculum demonstrates that fidelity and facilitator rapport with youth are associated with increases in student knowledge [24].

Contrary to our expectation, higher ratings of general facilitator characteristics at time 2 were associated with participants achieving less change in BMI. One possibility for this finding is that facilitators recognized that children in their group were struggling and subsequently increased their efforts with regard to the non-specific facilitator characteristics measured in this rating. Alternatively, this finding may have resulted from measurement error and/or facilitator reactivity to in-vivo observation. In particular, facilitators with group members who were not doing as well with regard to weight loss may have increased their efforts in response to the presence of observers.

A number of limitations need to be taken into account in considering the study findings. First, the nature of the study prohibited use of videotaped observations. As a result, facilitators were aware of observers within the group meetings and we were unable to calculate inter-rater reliabilities for observations. To alleviate potential reactivity to in-vivo observation, facilitators were assured that ratings would not be shared with YMCA staff and that ratings would be anonymous at the data entry phase. In addition, observers remained in the background to minimize any impact on group participants. Despite these limitations, differences in adherence were related to weight change, suggesting that reactivity of observations may not have been a major concern. A second limitation is that we used a modified measure of intervention fidelity. While standardized measures of intervention fidelity for certain interventions (e.g. motivational interviewing) do exist [25], we did not utilize a standardized measure because this type of measure does not exist for community-based behavioral weight control. Inclusion of a standardized measure with evidence of reliability and validity would have strengthened this study. Finally, this study did not include pre- and post-tests related to facilitator content knowledge included in the training. Examining changes in facilitator knowledge would be helpful for informing refinement of facilitator training practices. It is also important to recognize that adherence to content reflects only one aspect of a complex process through which content delivery, and participant knowledge acquisition and learning, are related to behavior change [26].

## Conclusions

Despite these limitations, this study provides important information. Our data suggest that paraprofessionals without prior expertise in pediatric weight control can be trained to successfully deliver an intervention that is evidenced based and incorporates behavioral and educational components. The observed adherence was even more impressive given the fact that facilitators had to manage the challenges of parents and children attending the same group session. Given the need for continued development and implementation of weight control interventions in community settings, these findings have important implications. Second, our findings suggest that investing time in training facilitators to adhere to a given protocol is critical and of higher priority than focusing on more general facilitator characteristics. It is noteworthy that even with high rates of content adherence, differences in adherence were related to changes in weight status. Just as models of learning [26] emphasize the importance of analyzing and applying knowledge in the process of acquiring knowledge, it is important to consider how paraprofessionals trained to deliver the intervention may have utilized additional strategies past simply verbalizing the curriculum (e.g., applying intervention content to the unique children/parents in their groups). Future research should include videotaped or audiotaped session observations to allow for assessment of observer reliability as well as consider the potential for identifying less intensive training models that result in equally high levels of facilitator adherence. In addition, it would be valuable to move beyond evaluation of content adherence to examine both participant and facilitator understanding of content (and ability to apply content to real-life situations) as related to behavioral outcomes.

## Competing interests

Elissa Jelalian has no financial disclosures.

Gary Foster is a consultant for UnitedHealth Group.

Amy Sato has no financial disclosures.

Kristoffer Berlin has no financial disclosures.

Cynthia McDermott is employed by the YMCA of Greater Providence.

Deborah Sundal is employed by UnitedHealth Group.

## Authors’ contributions

EJ contributed to the study design, data analysis and interpretation, and was primarily responsible for drafting the manuscript. GF contributed to study conception and design, interpretation of data, and assisted with drafting the manuscript. AS contributed to data acquisition, data analysis, and assisted with drafting the manuscript. KB performed the statistical analyses and contributed to drafting the manuscript. CM assisted with data acquisition and contributed to drafting the manuscript. DS contributed to study conception, data interpretation, and assisted with drafting the manuscript. All authors read and approved the final manuscript.
